# Case report: Optical coherence tomography for monitoring biologic therapy in psoriasis and atopic dermatitis

**DOI:** 10.3389/fmed.2022.995883

**Published:** 2022-09-27

**Authors:** Linh Ha-Wissel, Handan Yasak, Robert Huber, Detlef Zillikens, Ralf J. Ludwig, Diamant Thaçi, Jennifer E. Hundt

**Affiliations:** ^1^Department of Dermatology, Allergology and Venereology, University Hospital Schleswig-Holstein Lübeck (UKSH), Lübeck, Germany; ^2^Institute for Inflammatory Medicine, University of Lübeck, Lübeck, Germany; ^3^Institute of Biomedical Optics, University of Lübeck, Lübeck, Germany; ^4^Lübeck Institute of Experimental Dermatology (LIED), University of Lübeck, Lübeck, Germany

**Keywords:** optical coherence tomography (OCT), skin inflammation, psoriasis, atopic dermatitis, biologic therapy, *in vivo* imaging

## Abstract

Biologic therapies are increasingly used to treat chronic inflammatory skin diseases such as psoriasis and atopic dermatitis. In clinical practice, scores based on evaluation of objective and subjective symptoms are used to assess disease severity, leading to evaluation of treatment goals with clinical decisions on treatment initiation, switch to another treatment modality or to discontinue current treatment. However, this visual-based scoring is relatively subjective and inaccurate due to inter- and intraobserver reliability. Optical coherence tomography (OCT) is a fast, high-resolution, *in vivo* imaging modality that enables the visualization of skin structure and vasculature. We evaluated the use of OCT for quantification and monitoring of skin inflammation to improve objective assessment of disease activity in patients with psoriasis and atopic dermatitis. We assessed the following imaging parameters including epidermal thickness, vascular density, plexus depth, vessel diameter, and vessel count. A total of four patients with psoriasis or atopic dermatitis were treated with biologic agents according to current treatment guidelines. OCT was used to monitor their individual treatment response in a target lesion representing disease activity for 52 weeks. Psoriatic and eczema lesions exhibited higher epidermal thickness, increased vascular density, and higher vessel count compared to uninvolved skin. An upward shift of the superficial vascular plexus accompanied by smaller vessel diameters was seen in psoriasis in contrast to atopic dermatitis, where larger vessels were observed. A response to biologic therapy was characterized by normalization of the imaging parameters in the target lesions in comparison to uninvolved skin during the observation period of 52 weeks. Optical coherence tomography potentially serves as an instrument to monitor biologic therapy in inflammatory skin diseases. Imaging parameters may enable objective quantification of inflammation in psoriasis or atopic dermatitis in selected representative skin areas. OCT may reveal persistent subclinical inflammation in atopic dermatitis beyond clinical remission.

## Introduction

Psoriasis and atopic dermatitis are both common inflammatory skin diseases. About 30% of the patients with psoriasis (1) and about 10% with atopic dermatitis (2) require a systemic therapy due to their disease severity. Due to their safety profile and efficacy, biologic agents (3–5), and small molecules (6) are being increasingly used. A main drawback of these innovative treatments is that they may lose efficacy over time and that not all patients respond to a selected therapy. Also, in some cases the current dosing is not needed and potentially exposes the individuals to adverse events that may be avoided by adjusted dosing that fits their individual needs. Currently, however, indication and continuation of treatment are mainly dependent on clinical scores. These clinical scores are based on visual examination that might be biased by the experience of the clinician resulting in inter- and intraobserver variabilities (7, 8).

Facing these limitations, we aimed to develop an objective and more reliable evaluation method using image-based scoring to enable objective guidance of clinical decision making to avoid delayed and inconsistent therapy decisions.

Optical coherence tomography (OCT) is a fast, high-resolution, *in vivo* imaging method with growing influence in the dermatological practice, especially in non-melanoma skin cancer (9–12). The imaging technique of OCT is based on Michelson interferometry (13). In the assessment of inflammatory skin diseases, additional information on vascular network is of special interest. OCT angiography based on speckle variance detection allows the visualization of cutaneous vasculature (14). It was reported that different vascular patterns and shapes (dots, blobs, coils, lines, curves, and serpiginous vessels) could distinguish healthy skin from lesional inflammatory skin (15, 16). In psoriatic skin, structural changes and alterations in vessel density and size were shown (17–19). Previous studies demonstrated the correlation between histopathological findings and structural features of psoriasis and chronic inflammation apparent in OCT (20, 21). In atopic dermatitis, epidermal hypertrophy and vascular depth were discussed as important parameters for disease severity (22).

In the past 20 years, OCT was used for single time-point observations and short-term monitoring of psoriasis and atopic dermatitis (23–25). Based on these observations, here, we performed an interim analysis of a large ongoing prospective, long-term, observational study using a clinically approved OCT system for monitoring patients with psoriasis and atopic dermatitis undergoing biologic treatment over 52 weeks.

## Materials and methods

### Subjects and biologic therapies

**FIGURE 1:**
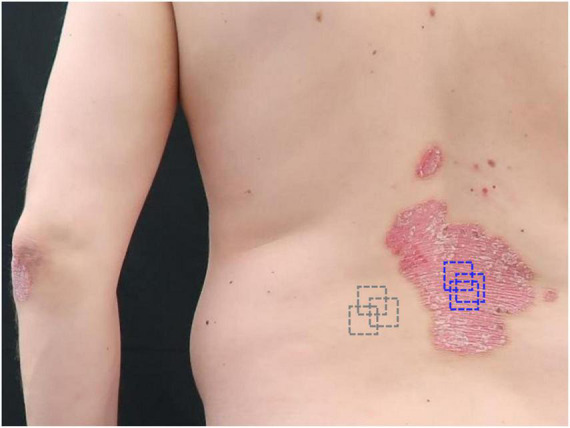
Selection of target lesions and control sites for optical coherence tomography (OCT). Patient 3 was a 45-year-old male patient with plaque psoriasis showing PASI = 10.8 and BSA = 10% at baseline. A representative target lesion (blue) on his trunk was selected for 52 weeks monitoring. A perilesional location was selected as the control site (gray). The scans were performed repetitively at the same lesional and non-lesional location.

The study was conducted according to the guidelines of the Declaration of Helsinki and ethical approval was obtained from the Ethics Committee of the University of Lübeck. Adult subjects eligible for inclusion were diagnosed with moderate to severe plaque psoriasis or atopic dermatitis and received treatment according to current treatment guidelines. Written informed consent was obtained from all patients. OCT data of three patients with psoriasis and one patient with atopic dermatitis was analyzed. They underwent biologic treatment with anti-IL-17 ixekizumab (*n* = 1), anti-IL-23 risankizumab (*n* = 1), anti-TNF-α certolizumab (*n* = 1) or anti-IL-4Rα dupilumab (*n* = 1). At baseline, week 2, 4, 16, 28, 40, and 52 of treatment, clinical scores were determined and OCT scans were performed at one lesional site and one perilesional control site for each patient (Figure 1).

### Clinical scoring

Visual-based scores such as psoriasis area and severity index (PASI) (7), eczema area and severity index (EASI) (8), and body surface area (BSA) (7) were determined by an experienced physician. Additional digital dermoscopy images were captured with DermoGenius ultra polarized (DermoScan GmbH, Regensburg, Germany).

To date, whole body OCT scanners do not exist and large-area scanning cannot be performed within a reasonable time. Thus, we must select target lesions for small-area imaging. The total sign score (TSS) was used to evaluate selected skin areas. For psoriasis, three clinical signs including erythema, induration, and desquamation were assessed using a 5-point scale (0 absent, 1 mild, 2 moderate, 3 severe, and 4 very severe) with a total range of 0–12 (26). For atopic dermatitis, six clinical signs including erythema, edema/papulation, oozing/crusting, excoriation, lichenification, and dryness were graded using a 4-point scale (0 absent, 1 mild, 2 moderate, and 3 severe) with a total range of 0–18 (27).

### Optical coherence tomography

We utilized a clinically approved VivoSight Dx OCT scanner (Michelson Diagnostics, Maidstone, Kent, UK). The emission wavelength is 1,305 nm. The lateral and axial resolution is <7.5 μm and <5 μm, respectively. The scan area is 6 mm × 6 mm. The penetration depth is about 1.2 mm. A three-dimensional image stack with 6 mm width records a sequence of 180 B-scans with an interslice spacing of 33.3 μm. Images were acquired as vertical B-scans and *en-face* scans. The acquisition time of an image stack is about 30 s generating both structural and vascular images. The *en-face* scans are reconstructed images to show skin layers at a constant depth from the skin surface. For an image stack with 1.2 mm depth, a sequence of 120 *en-face* scans with an interslice spacing of 10 μm can be obtained. Patients were at rest prior to scanning. Measurement conditions (location, patient position, and room temperature) were kept constant. Three regions of the same target lesion and three regions of its perilesional, clinically uninvolved skin area as reference were repetitively imaged. The target lesion was located either in upper extremities or trunk. Comparable to conventional computer-assisted dermoscopy, we used system-integrated overview images of the body and skin areas to make sure that measurements are done in consistent areas. The laser scanning handpiece uses a plastic tip as a distance holder between optical component and skin surface. The distance holder was gently placed onto the skin to avoid pressure- or shear-induced effects on the vessel diameter (28). Terminal hair was carefully trimmed. Scales were not removed. According to the patients’ verbal feedback, the OCT scans do not lead to any patient discomfort.

### Imaging parameters

**FIGURE 2:**
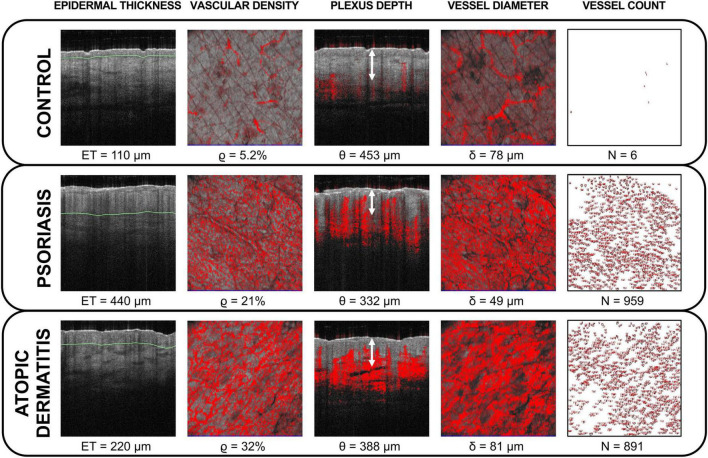
Optical coherence tomography (OCT) imaging parameters for objective quantification of skin inflammation. Epidermal thickness (*ET*), vascular density *ϱ*, plexus depth θ, vessel diameter δ, and vessel count *N* were calculated in unaffected skin (control) and in target lesions (psoriasis and atopic dermatitis) at baseline. Lesional inflammatory skin exhibited increased epidermal thickness denoted by green lines and alterations in vasculature in comparison to control sites. The vascular density is exemplarily shown at 400 μm depth and the vessel diameter is shown at 600 μm depth. The plexus depth is indicated by white arrows. The vessel count was performed at 200 μm depth.

Clinical signs such as erythema and skin thickening can result from microvascular changes and epidermal hypertrophy, respectively. Thus, we defined epidermal thickness, vascular density, depth, diameter, and vessel count as imaging parameters for quantification of skin inflammation (Figure 2). The calculation of vascular density, depth, and diameter was assisted by the proprietary vessel analysis software (Michelson Diagnostics, Maidstone, Kent, UK). All measures were performed by the same operator.

#### Epidermal thickness *ET*

We used the integrated on-screen ruler tool to measure the maximum epidermal thickness (*ET*) per image stack, then a mean ET of three image stacks was calculated with standard deviation. *ET*_*L*_ is the epidermal thickness of the target lesion and *ET*_*C*_ of the control at baseline. The same indices were used analogously for the other imaging parameters.

#### Vascular density *ϱ*

The vascular density *ϱ* was calculated at the depth of the superficial plexus. It is the density of the top of the superficial plexus calculated over the depth range, which is the plexus depth ± 60 μm.

#### Plexus depth *θ*

The plexus depth θ is the depth of the top of the superficial plexus, where the density reaches 50% of the maximum vessel density.

#### Vessel diameter *δ*

The vessel diameter δ is a modal value calculated at the depth of the superficial plexus. It is the diameter of the majority of the vessels.

#### Vessel count *N*

The vessel count *N* was calculated using ImageJ 1.53k (U.S. National Institutes of Health, Bethesda, MD, USA). We anticipated that the cross sections of the elongated capillaries could be most accurately measured at about 200 μm depth, as the last few *en-face* scans capture undesired projection artifacts from the superficial vessels. The *en-face* images were thresholded and converted into binary images. Adjacent or overlapping vessels were divided by watershed segmentation. The vessels were counted by automatic particle analyzer using Sobel edge detection.

### Response rate

We obtained fit coefficients λ using MATLAB R2021b (The MathWorks, Natick, MA, USA) to fit *ET* to an exponential equation *ET* = *A*×exp(−λ×*t*)+*ET*_*R*_ as a function of time *t* based on least squares algorithm, where *ET*_*R*_ is the estimated minimum *ET* during 52 weeks of therapy and *A* is an adjusting parameter. The number of weeks of therapy *t*_*R*_ required for *ET* reduction achieving 1.25 × *ET*_*R*_ is then given by *t*_*R*_ = −λ^−1^×*ln*[(*k*×*ET*_*R*_−*ET*_*R*_)/*A*] for *k* = 1.25. If physiologically achievable, instead of *k* × *ET_*R*_*, *t*_*R*_ can also be calculated for 0.5 × *ET_*L*_* (50% reduction of *ET*_*L*_ from baseline).

### Statistical analysis

We used Pearson correlation coefficient *r* to show the correlation between *ET* or *N* and the clinical scores.

## Results

### Description of patients at baseline

Patient 1 was a 52-year-old male with psoriasis (PASI = 19.5, BSA = 37%) who was treated with ixekizumab. He had no previous systemic therapy.

Patient 2 was a 30-year-old male with psoriasis (PASI = 8.7, BSA = 11%) who received risankizumab. Prior therapies were acitretin, dimethyl fumarate, methotrexate, certolizumab, ustekinumab, and secukinumab.

Patient 3 was a 45-year-old male with psoriasis (PASI = 10.8, BSA = 10%) who underwent therapy with certolizumab. He was previously treated with methotrexate.

Patient 4 was a 50-year-old female with atopic dermatitis (EASI = 17, BSA = 25%) who was treated with dupilumab. She had no previous systemic therapy.

In patient 1 and 2, the target lesions were located on their right arms. In patient 3 and 4, the target lesions were located on their trunk. The control area was their perilesional skin.

### Structural imaging parameters

#### Epidermal thickness

**FIGURE 3:**
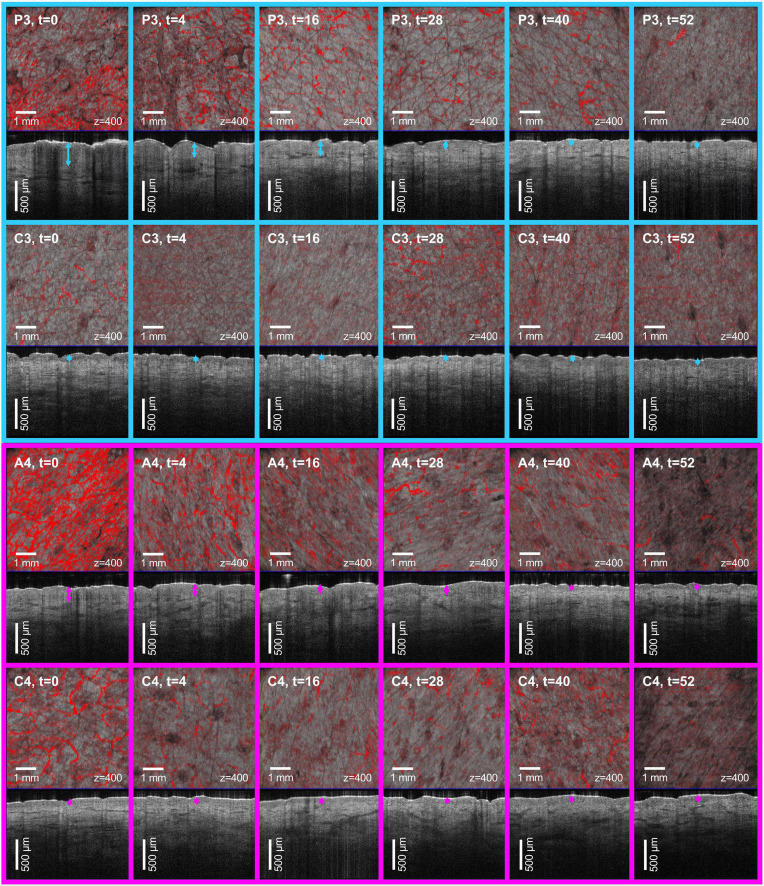
Optical coherence tomography (OCT) guided monitoring of biologic therapy in psoriasis and atopic dermatitis. Patient 3 with psoriasis (P3) was treated with certolizumab (top) and patient 4 with atopic dermatitis (A4) was treated with dupilumab (bottom) for *t* = 52 weeks. The vertical B-scans and the corresponding *en-face* scans at 400 μm depth were collected in the course of therapy. Hyperkeratotic psoriatic plaques led to a signal reduction at baseline. Under therapy a decrease of the epidermal thickening (arrows) and a normalization of the vascularization (red signal) were observed in psoriasis and atopic dermatitis. No significant changes were detected in the control sites (C3 and C4).

**FIGURE 4:**
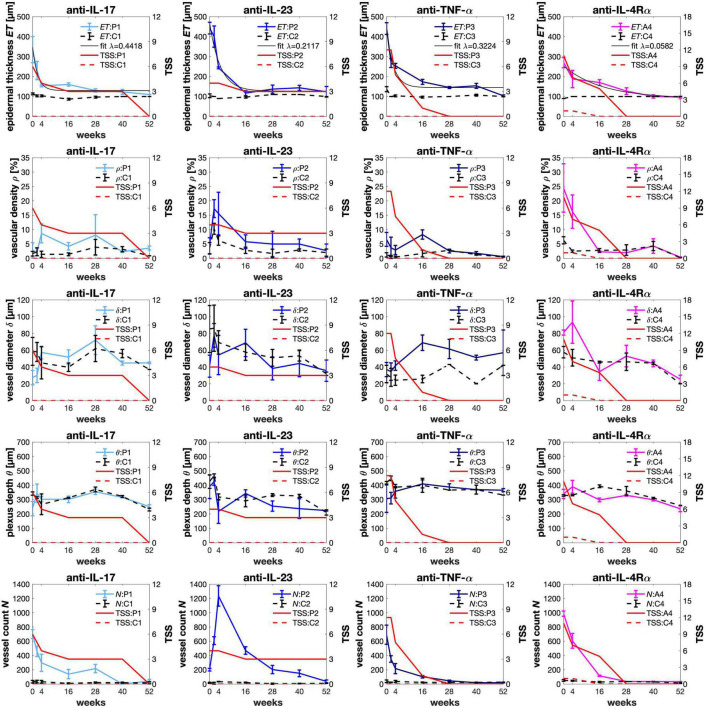
Monitoring therapy response using optical coherence tomography (OCT). In psoriasis (P1–P3) and atopic dermatitis (A4), the epidermal thickness (*ET*) was fitted to calculate the exponential decay rate or response rate λ. The vessel count *N* was calculated using ImageJ (U.S. National Institutes of Health, Bethesda, MD, USA). After 52 weeks of treatment, we observed a full normalization of the increased epidermal thickness and the vessel count. The vascular parameters such as vascular density *ϱ*, vessel diameter δ, and plexus depth θ were calculated by using the proprietary VivoSight vessel analysis software (Michelson Diagnostics, Maidstone, Kent, UK). Prior to treatment, the vascular density in inflamed skin was higher than in the control areas. Interestingly, we observed an upward shift of the plexus depth and smaller vessel diameters in psoriatic skin in comparison to the control at baseline. Eczematous skin showed larger vessel diameters. As a potential sign of therapy response, the vascular parameters normalized during therapy course. For correlation, the total sign score (TSS) was used to clinically assess the target lesions and their control sites. For psoriasis, erythema *E*, induration *I*, and desquamation *D* were assessed using a 5-point scale (0–12). For atopic dermatitis, six clinical signs including erythema *E*, papulation *I*, excoriation *Ex*, lichenification *Li*, crusting *C*, and dryness *Dr* were graded using a 4-point scale (0–18).

In line with clinical and histopathological findings, at baseline we measured a thicker *ET*_*L*_ in psoriasis due to hyperkeratosis (Figure 3, top). A similar observation was made in atopic dermatitis where the increased *ET*_*L*_ was due to spongiosis and/or lichenification (Figure 3, bottom). At baseline, the mean *ET*_*L*_ was 405.8 μm (±20.8 μm) in psoriatic skin and 270 μm (±25.5 μm) in atopic dermatitis. *ET*_*C*_ ranged from 90 to 110 μm and was similar in uninvolved skin of patients with psoriasis and atopic dermatitis. The thickened *ET*_*L*_ decreased during the observation period under therapy (Figure 4, row 1). According to *t*_*R*_, 0.5 × *ET_*L*_* was achieved as follows: ixekizumab (after 3.95 weeks), certolizumab (after 4.23 weeks), risankizumab (after 6.08 weeks), and dupilumab (after 22.13 weeks). 1.25 × *ET_*R*_* was achieved as follows: ixekizumab (after 4.31 weeks), certolizumab (after 6.52 weeks), risankizumab (after 11.23 weeks), and dupilumab (after 33.89 weeks).

In good agreement with the OCT measurements, the clinical severity was reduced in the target lesion (TSS) and in the global assessment (PASI or EASI) (Supplementary Figure 1). A positive correlation was observed between *ET*_*L*_ and clinical scores as follows: ixekizumab (*r_*TSS*_* = 0.83, *p* = 0.0208; *r_*PASI*_* = 0.9564, *p* = 0.0007), certolizumab (*r_*TSS*_* = 0.8857, *p* = 0.008; *r_*PASI*_* = 0.9265, *p* = 0.0027), risankizumab (*r_*TSS*_* = 0.8968, *p* = 0.0062; *r_*PASI*_* = 0.8017, *p* = 0.0301), and dupilumab (*r_*TSS*_* = 0.9778, *p* = 0.0007; *r_*EASI*_* = 0.9184, *p* = 0.0097).

#### Decay rate

The decrease of *ET*_*L*_ under therapy yielded the following decay rates: ixekizumab (λ = 0.4418, *R*^2^ = 0.96), certolizumab (λ = 0.3224, *R*^2^ = 0.95), risankizumab (λ = 0.2117, *R*^2^ = 0.96), and dupilumab (λ = 0.05817, *R*^2^ = 0.93). In this comparison, the highest rate was observed for ixekizumab followed by certolizumab and risankizumab. The decay rate under dupilumab was a factor of 3.6–7.6 slower than the biologic therapies of psoriasis.

### Vascular imaging parameters

#### Vascular patterns and shapes

We used the terminology as proposed by Ulrich et al. (15). Characteristic vascular patterns and shapes were visible in the *en-face* mode at about 200 μm depth (Supplementary Figure 2). Psoriatic skin exhibited “dotted” or “pinpoint-like” vessels resulting from vessel elongation within extended dermal papillae. Lichenification is often observed in chronic eczema. Coarsening and wrinkling of the lichenified skin may change the direction of the capillary loops causing a striped pattern. Thus, “comma-like” vessels were observed in atopic dermatitis. Uninvolved skin typically has “linear” vessels generating a reticular pattern.

#### Vascular density, plexus depth, and vessel diameter

Vascular parameters decreased or normalized during therapy course (Figure 4, rows 2–4). Before treatment the mean *ϱ_*L*_* in psoriatic skin was 4.6% (±1.9%), which was higher than *ϱ_*C*_* with 2.1% (±1.23%). *ϱ_*L*_* in atopic dermatitis was 24.5% (±8.4%), while *ϱ_*C*_* was 6.3% (±1.24%). The higher *ϱ_*L*_* from baseline decreased after therapy start as a result of reduction in vessel diameter and/or vessel count (Supplementary Figure 3).

We defined the location of the origin (0,0) in the upper left of the image according to the general convention in image processing. This means that plexus depth and optical axis *z* have the same direction. For plots, the origin is located in the lower left. We observed an upward shift of the plexus Δθ = θ_*L*_-θ_*C*_ by −83.4 μm (± 42.3 μm) in psoriatic skin θ_*L*_ when compared to θ_*C*_. At baseline, the mean δ_*L*_ in psoriatic skin was 35.2 μm (± 6.9 μm), which was smaller than δ_*C*_ with 58.6 μm (±26.8 μm). We did not observe an upward shift in the patient with atopic dermatitis. In contrast to the psoriatic lesions, in atopic dermatitis δ_*L*_ was 81 μm (±3.1 μm), which was larger than δ_*C*_ with 57.7 μm (±6.9 μm).

#### Vessel count

The mean *N*_*L*_ in psoriasis and atopic dermatitis was 531.4 (±285.9) and 987 (±37.5), respectively. The mean *N*_*C*_ was 35.08 (±14.27). After 52 weeks, we observed a full normalization of the vessel count (Figure 4, row 5).

Also, a positive correlation was observed between *N* and clinical scores as follows: ixekizumab (*r_*TSS*_* = 0.85, *p* = 0.0154; *r_*PASI*_* = 0.9589, *p* = 0.0006), certolizumab (*r_*TSS*_* = 0.8978, *p* = 0.0061; *r_*PASI*_* = 0.9327, *p* = 0.0022), risankizumab (*r*_*TSS*_ = 0.6055, *p* = 0.1497; *r*_*PASI*_ = 0.6792, *p* = 0.0933), and dupilumab (*r*_*TSS*_ = 0.9378, *p* = 0.0057; *r*_*EASI*_ = 0.9272, *p* = 0.0078).

### Clinical response

The results of this case series indicated a good correlation with the clinical scores. A 75% reduction from baseline in the TSS (TSS-75) was achieved under ixekizumab after 3 weeks and under certolizumab after 3.5 weeks. In this observation period, TSS-75 under risankizumab was not achieved. After 3 weeks, TSS-75 was achieved under dupilumab.

## Discussion

Optical coherence tomography is a suitable imaging tool for the investigation and monitoring of inflammatory skin diseases. The VivoSight OCT offers user-friendly handling and fast scanning, which is important for its application as a bedside monitoring device. But the system applied in this study has a limited resolution capacity that does not allow the detection of single cells. In comparison, the investigational line-field confocal OCT provides superior axial resolution of 1.1 μm and lateral resolution of 1.3 μm (29). This would allow a characterization of individual cells and microstructures in inflammatory skin diseases (30). Furthermore, OCT with dynamic contrast allows color-coding of different epithelial cell layers based on micromotions of cellular structures (31, 32). So far, these technical upgrades have not yet been incorporated into a clinically approved OCT system for dermatological applications. The future goal is to obtain contactless, whole body scans *via* rapid scanning of large body sites as commercial OCT systems only provide small fields of view (33, 34). To date, we are dependent on the selection of target lesions for imaging. While psoriatic lesions tend to recur at the same skin sites that were affected previously, in atopic dermatitis the eczema lesions tend to shift location. Under this aspect, monitoring of target lesions could potentially miss an eczema flare or even psoriasis that exacerbates on a different body part.

Psoriasis and atopic dermatitis are associated with lower skin hydration (35, 36). Consequently, effects of laser-tissue interactions such as scattering (e.g., scaling, hyperkeratosis, and lichenification) and shielding (e.g., shadow artifacts caused by hair or crusts) could lower optical penetration and might masquerade as a loss of perfusion.

In an attempt to define imaging biomarkers for inflammatory skin diseases, we demonstrated the application of OCT for monitoring biologic therapy in psoriasis and atopic dermatitis based on four case studies. Biomarkers are objective, quantifiable, and reproducible (37). We should differentiate between robust and weak imaging biomarkers. Robust biomarkers might be *ET* and *N*, as these parameters are less influenced by internal and external factors. In this study, manual measurements of the maximum *ET* still remain vulnerable to intraobserver variability, which was minimized by repeated measurements. Hence, further studies should incorporate computer-assisted analysis of the *ET*. Weaker biomarkers such as *ϱ* and δ might be highly influenced by internal stress factors of the subject (comparable to the “white coat effect”) and outdoor temperatures (hot temperatures lead to vasodilation and cold temperatures lead to vasoconstriction) as we observed intraindividual variations in control sites.

In this work, we provided a detailed description on vascular alterations in psoriasis and atopic dermatitis compared to non-lesional skin. Our findings were consistent with previous observations reported on higher vascularization and characteristic vascular pattern in psoriasis (16–18). While “dotted” vessels were previously described in psoriasis (38), we firstly described the appearance of “comma-like” vessels in atopic dermatitis with lichenification. We detected an upward shift of the plexus in psoriasis. We anticipated that a plexus shift results from an elongation of the capillary loops, which is more distinctive in severe psoriasis. At baseline, no shift of the plexus depth was observed in our patient with atopic dermatitis. Byers et al. (22) described deeper vascular layers in atopic dermatitis so that we need to make further investigations before coming to a conclusion. We observed large vessels and high vascular densities in atopic dermatitis that were consistent with former studies (38, 39). Manfredini et al. (23) reported on a reduction of dermal edema and vascularization under dupilumab. In our analysis, we also observed a normalization of the vasculature under therapy. Further, we observed that vessel elongation in psoriasis appeared with smaller capillary loop diameters. Evidently, a larger case number is required to confirm our preliminary observations.

The aim of biologic treatment especially in atopic dermatitis is to improve the skin barrier. We observed a good correlation between *ET* and TSS. Our results on the decrease of *ET* under therapy were in good agreement with previous studies using topical (25, 40) and non-biologic systemic therapies (19). In contrast to conventional therapies of psoriasis, we observed an accelerated decrease of *ET*. Similar results on *ET* in atopic dermatitis were reported under dupilumab (23). We also anticipated that *ET* may be a robust imaging biomarker as already stated by Byers et al. (22). The rapid decrease in *ET* can be regarded as an exponential decay. We interpreted the decay rate λ as a response rate. In theory, λ = 0 means steady state. Values of λ > 0 implicate therapy response meaning the larger λ the higher the response. λ < 0 refers to therapy failure.

Patient 2 was bio-experienced with a long record of pretreatments, therefore, a slower therapy response in TSS was seen under risankizumab. Interestingly, in patient 4 with atopic dermatitis higher vessel density and diameter were observed in the control area at baseline indicating subclinical inflammation in clinically healthy-appearing skin. In addition, although a rapid improvement of patient 4 was clinically observed shown as TSS-75 after 3 weeks, using OCT we were able to detect a prolonged epidermal thickening for *t*_*R*_(1.25 × ET_*R*_) = 33.89 weeks as a sign of persistent disease activity. Comparable observations were reported by Byers et al. (22). The understanding of subclinical inflammation highlights the importance of therapy continuation to avoid the risk of relapse (41).

## Conclusion

Preliminary observations of this work showed that OCT may be suitable for objective quantification of structural (epidermal thickness) and vascular parameters (vascular density, depth, diameter, and count). These parameters may serve as objective imaging biomarkers for monitoring therapeutic effects in psoriasis and atopic dermatitis. The relatively short acquisition time of OCT is an important demand for medical imaging to minimize time burden for patients.

Therapy response may be characterized by reduction of epidermal thickness and normalization of vascular network. Potential imaging biomarkers such as epidermal thickness and vessel count exhibited rapid changes. The calculated response rate λ may serve as a useful parameter in the assessment of therapeutic effects. Additional diagnostic value of OCT angiography could be seen in the detection of subclinical inflammation that implicates the need for therapy continuation beyond clinical remission.

In this preliminary evaluation, all patients have responded well to their systemic treatment. In the next step, we will also evaluate insufficient therapy responses or therapy failures in a large prospective, long-term, observational study. Additional imaging biomarkers revealing desquamation, excoriation, and lichenification should be considered in upcoming studies. The vision of the future is to provide “OCT-guided therapy” enhancing the current dermatological assessment and contributing to personalized medicine.

## Data availability statement

The original contributions presented in this study are included in the article/Supplementary material, further inquiries can be directed to the corresponding author.

## Ethics statement

The studies involving human participants were reviewed and approved by Ethics Committee of the University of Lübeck, Germany. The patients/participants provided their written informed consent to participate in this study.

## Author contributions

LH-W and HY contributed to data acquisition. LH-W performed the data analysis and wrote the main manuscript text. All authors contributed to manuscript revision, read, and approved the submitted version.
