# Supplementary material for: Case report: Optical coherence tomography for monitoring biologic therapy in psoriasis and atopic dermatitis
Source: Front Med (Lausanne). 2022 Sep 27;9:995883. doi: 10.3389/fmed.2022.995883 (PMC9551172; doi:10.3389/fmed.2022.995883)
Supplement: Supplementary file 1 [file Data_Sheet_1.pdf]

Supplementary Material

1 Supplementary Figures

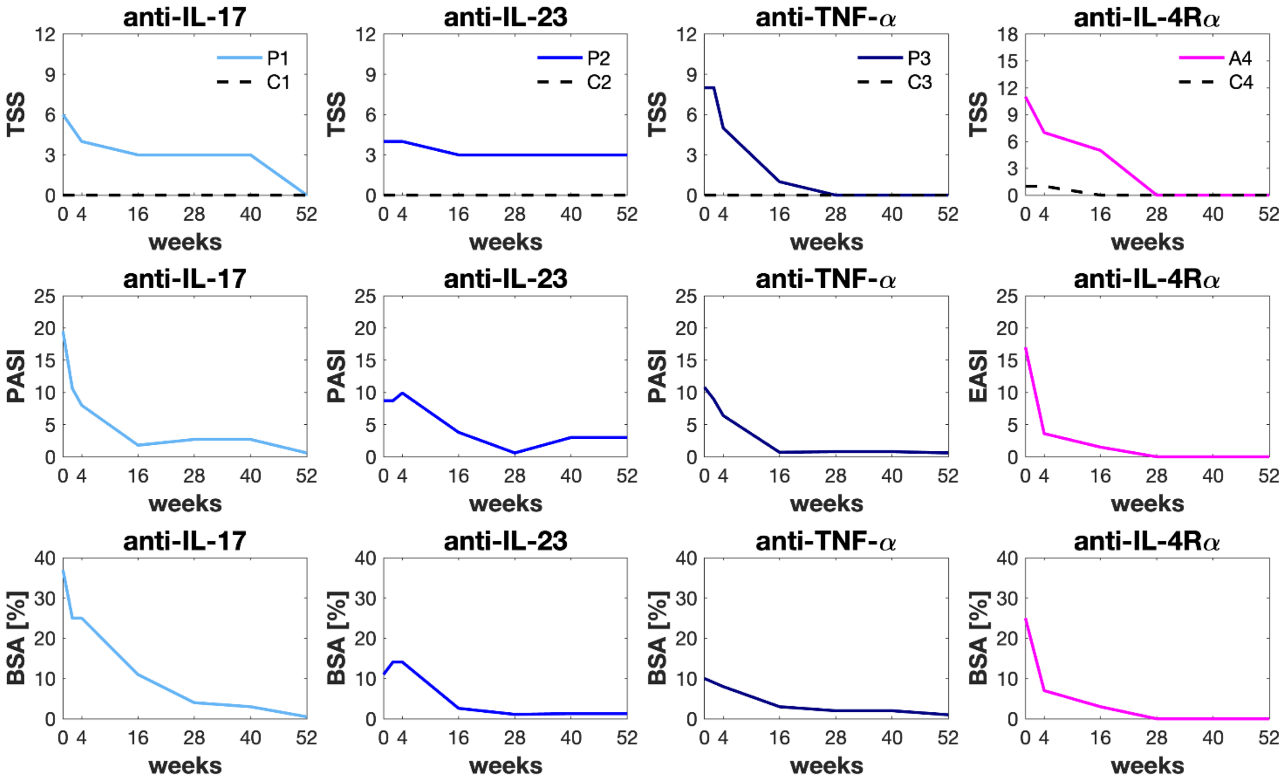

Supplementary Figure 1. Clinical scoring systems. The total sign score (TSS) in target lesions and non-lesional control sites (C1-C4) was determined for psoriasis (P1-P3) with a total range of 0-12 and for atopic dermatitis (A4) with a total range of 0-18. Global scores such as psoriasis area and severity index (PASI) and eczema area and severity index (EASI) were also determined as the gold standard with a total range of 0-72. The body surface area (BSA) gives the total percentage of the affected body surface. Therapy response was indicated by a decrease of these clinical scores.

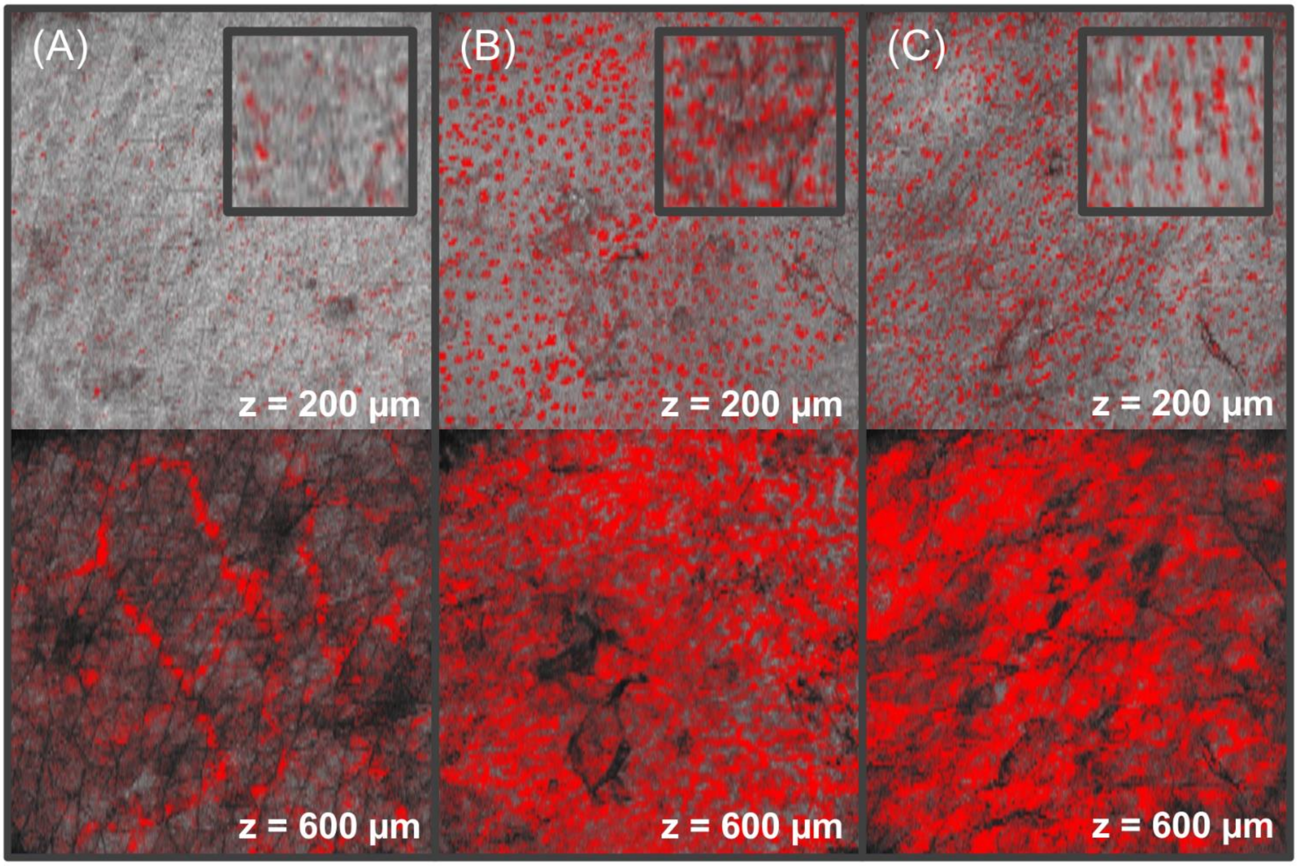

Supplementary Figure 2. Vascular patterns and shapes in optical coherence tomography. The horizontal *en-face* scans displayed different tissue depths *z* for **(A)** non-lesional control site, **(B)** plaque psoriasis (patient 2), and **(C)** atopic dermatitis (patient 4). At about 200 µm depth, characteristic vascular patterns and shapes of skin inflammation were visible. Psoriatic skin exhibited “dotted” or “pinpoint-like” vessels. Skin with atopic dermatitis exhibited “comma-like” vessels. Underlying deeper tissue layers showed a higher vascular density owing to the inflammatory processes. The “reticular” pattern of the superficial vascular plexus in non-inflamed skin at 600 µm depth was hardly recognizable in psoriasis and atopic dermatitis at the same tissue depth. Terminology to describe vessel morphology was used according to Ulrich *et al*.[15]

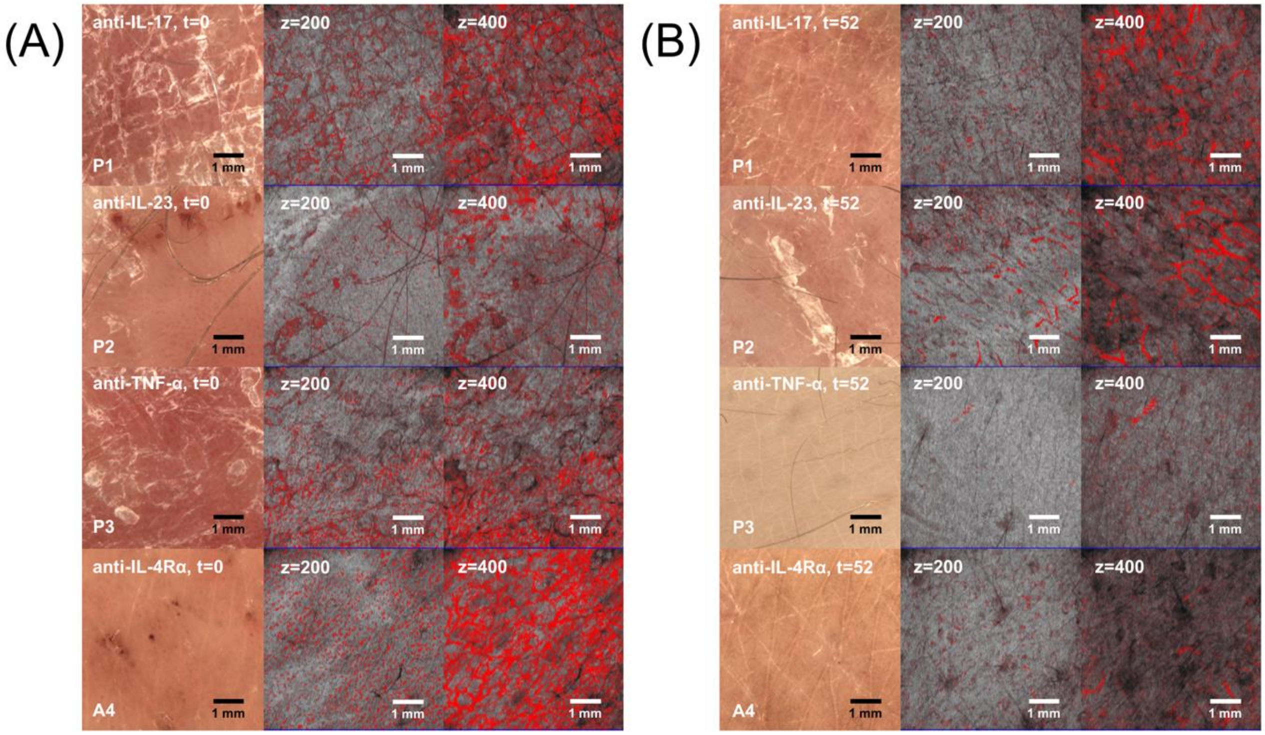

Supplementary Figure 3. Dermoscopy and en-face OCT images of psoriasis and atopic dermatitis. Dermoscopy images show the clinical appearance of the inflammatory skin lesions in patients with psoriasis (P1-P3) and atopic dermatitis (A4). The underlying vascular network is visualized by *en-face* scans at 200 µm and 400 µm depth. **(A)** At $t = 0$ weeks (baseline) a biologic treatment was initiated. **(B)** At $t = 52$ weeks (endpoint) the treatment outcome was evaluated.
